# How Much Do Strategy Reports Tell About the Outcomes of Neurofeedback Training? A Study on the Voluntary Up-Regulation of the Sensorimotor Rhythm

**DOI:** 10.3389/fnhum.2020.00218

**Published:** 2020-06-10

**Authors:** Miriam Autenrieth, Silvia E. Kober, Christa Neuper, Guilherme Wood

**Affiliations:** ^1^Institute of Psychology, University of Graz, Graz, Austria; ^2^BioTechMed-Graz, Graz, Austria

**Keywords:** sensorimotor rhythm, neurofeedback, strategy reports, non-responders, categorization

## Abstract

The core learning mechanisms of neurofeedback (NF) training are associative, implicit, and, consequently, largely impervious to consciousness. Many other aspects of training that determine training outcomes, however, are accessible to conscious processing. The outcomes of sensorimotor rhythm (SMR) up-regulation training are related to the strategies reported by participants. The classification methods of individual strategies employed hitherto were possibly under influence of the idiosyncratic interpretation of the rater. To measure and possibly overcome this limitation, we employed independent raters to analyze strategies reported during SMR up-regulation training. Sixty-two healthy young participants took part in a single session of SMR up-regulation training. After completing six blocks of training, in which they received either simple visual feedback or a gamified version thereof, participants were required to report the strategies employed. Their individual learning outcomes were computed as well. Results point out that individual strategies as well as NF learning outcomes were not particularly sensitive to the presence of gamified elements in training the SMR up-regulation. A high degree of consistency across independent raters classifying strategy reports was observed. Some strategies were more typical of responders while other ones were more common among non-responders. In summary, we demonstrate a more objective and transparent way to analyze individual mental strategies to shed more light on the differences between NF responders and non-responders.

## Introduction

In neurofeedback (NF), a person’s brain activity (e.g., the electroencephalogram - EEG) is recorded, analyzed, and presented in real time to participants to help them learn how to regulate their brain activity (Coben and Evans, 2010). Successful NF training can improve cognitive performance in healthy and clinical populations. Up-regulation of spectral power in the frequency band 12–15 Hz, as known as the sensorimotor rhythm (SMR), has been related to better outcomes in attention, short-term memory, and memory consolidation (Gruzelier, 2014; Sitaram et al., 2017). Despite its efficacy in the majority of participants, 15–30% of the participants are unable to control their own brain activity (Dickhaus et al., 2009). Although this so-called brain–computer interface (BCI) illiteracy has become a major problem in EEG NF research (Allison and Neuper, 2010), no satisfactory answer to the question of which factors determine the NF training success is available so far (Enriquez-Geppert et al., 2017). According to literature reviews on EEG NF (Gruzelier, 2014; Gaume et al., 2016; Sitaram et al., 2017) and EEG BCI (Jeunet et al., 2015), the number of non-responders observed after completion of training is generally in the interval 15–30%. In studies with only one or only a few sessions of SMR training, the number of non-responders uses to be higher than 15–30%.

Cognitive strategies may hamper or promote NF learning for they indicate how appropriate is the tuning of cognitive resources during NF learning (Gaume et al., 2016; Davelaar et al., 2018). Empirical studies show that mental strategies differ substantially in their effectiveness during NF training (Hardman et al., 1997; Hinterberger et al., 2005; Nan et al., 2012; Kober et al., 2013).

Kober et al. (2013) suggest that both the successful NF training and the choice of mental strategies are a result of automatization. A dual process theory (Lacroix et al., 1986) of NF learning suggests that at the beginning, a person tries various strategies and commits to the most effective one, improves and stores it as a heuristic in implicit memory. At some point, plastic reorganization of neural networks can render an originally successful heuristic obsolete. When this happens, participants may feel compelled to try new strategies, which, at least at the beginning, rely more on conscious processing (i.e., rule-based system) and become gradually more automatized and implicit (i.e., skill- or experience-based system) with practice (Dietrich, 2004; Gaume et al., 2016; Davelaar et al., 2018).

Nevertheless, automation alone may not be sufficient to explain the contents of strategy reports. If the reports on “No Strategy” were only a matter of automatization, one should expect that regardless of the brain waves being trained, responders in their majority would tend to report “No Strategy” after sufficient practice. While this is reported in SMR up-regulation training (Kober et al., 2013, 2017), in other brain rhythms other strategies lead to the best outcomes. Kober et al. (2013) reported that “Concentration” was the most successful strategy when training upregulation in a narrow Gamma band (40–43 Hz). Nan et al. (2012) reported that during individual alpha peak NF training, most successful thoughts were related to positive mood, namely thinking of “Friends,” “Love” and “Family,” while emotionally neutral strategies had limited success. Similarly, in the study by Zoefel et al. (2011), most subjects reported “Evoking Emotions” as the best strategy to up-regulate upper-alpha power. In contrast, when training the downregulation of alpha power (8–12 Hz), Ros et al. (2013) found “*focused visual attention*” to be the preferred strategy of over 70% of participants. These results suggest that strategy reports carry specific information about the brain signals being trained. According to Gaume et al. (2016), two basic skills determine the individual ability to learn from NF: the aptitude to achieve an inner perception of the brain signal (i.e., discrimination), as well as the ability to voluntarily modulate it in the intended direction (i.e., self-maintenance). These two skills are mobilized with the aim of constructing schemata, which are individual packages of cognitive actions aiming at maximizing positive feedback. One can expect the schema to be specifically related to the brain rhythms being trained and determine the strategies reported by individual participants, for this may explain why even after prolonged practice different brain rhythms do not all lead to reports of “No Strategy” but rather to other strategy preferences.

Respecting these strategy preferences also seems to be important to optimize learning. Hardman et al. (1997) investigated how instructing participants interferes with NF learning. These authors trained the ability to control hemispheric asymmetries in slow cortical potentials (SCP) using two sets of strategies. The first group of participants was oriented to employ positive emotional imagery to activate the left hemisphere and negative emotional imagery to activate the right hemisphere. The second group received less precise instructions (i.e., “*to use different brain areas*”) to solve the task. Participants instructed to use emotional imagery when modulating the hemispheric asymmetries in SCP training were less effective than participants free to find the optimal way to modulate their SCP signals. Evidence provided by Hardman et al. (1997) suggests that the effort to build up a useful “internal reference” (i.e., aschema) is larger when using mental imagery in SCP training than when participants are free to choose their strategy freely by trial-and-error. The results by Hardman et al. (1997) suggest that tailoring instructions for specific NF training protocols depends on providing both useful information for participants to get started as well as sufficient freedom to build up a useful internal reference. However, this is not the only possible interpretation of the results. Emotion imagery is not the most suitable strategy to induce SCP asymmetries and is disadvantageous for participants instructed to use it as in the Hardman et al. (1997). In a recent study, Kober et al. (2018b) observed that instructing participants to downregulate the SMR rhythm by “relaxing and focusing on the task” – instructions that induce SMR upregulation in most participants – disrupts their performance considerably.

Although many studies on individual mental strategies are available, only a few such study designs were conceived explicitly and specifically to examine the impact of mental strategies on training outcomes (Hardman et al., 1997; Hinterberger et al., 2005; Nan et al., 2012; Kober et al., 2013, 2017; Ros et al., 2013; Davelaar et al., 2018). Moreover, in previous studies strategies were classified using *post hoc* classification keys devised by the experimenters themselves (Hinterberger et al., 2005) using the contents of the individual reports. Interference of experimenters’ subjectivity in the classification of strategies may have contaminated previous studies since none of them employed independent raters to classify strategies or employed any other more objective method to classify the strategies.

Furthermore, the impact of different NF displays on strategy reports or training success was never evaluated, because a single NF display was employed in each study. The user experience with NF training is determined not only by the choice of strategies to guide performance, but also the learning environment and the kind of feedback provided, which are supposed to be engaging and motivating for the participant along the duration of training. Besides focusing on individual mental strategies, we also addressed the question of the impact of feedback design in the present study. Serious games have gained in importance increased in recent years in the design of NF training protocols. Serious games are games that have more goals than pure entertainment (Ninaus et al., 2014, 2015). It is about uniting all aspects of learning and offering this playful treatment to all age groups, and not just children and adolescents (Susi et al., 2007). Particularly in association with NF, the motivation and interest during training can be increased with enriched and game-like three-dimensional feedback modalities (Gaume et al., 2016; Kober et al., 2016, 2018a). Due to the scarcity of empirical studies, it remains open whether game-like feedback screens may improve NF training outcomes when compared to classic NF training settings (Kober et al., 2018a).

In the present study, we collected data of a larger number of participants to increase the accuracy of estimation of frequency of strategies. The NF training was presented in two different variants; in a classical version using bars and in a gamified version using a worm race to compare the training performance and strategy reports in both feedback screen conditions. Training consisted of one single session to focus on the early stage choice of strategies and investigate whether participants with positive or negative outcomes can be identified early by the kind of strategy they use. Strategy reports were collected after training. Participants were instructed “*to relax and focus on the task with the aim of increasing the height of the central bar whole keeping the lateral bars as low as possible.*” Strategy reports describe the individual interpretation/implementation of the task instructions given by the experimenter as perceived by each participant. Participants were asked to describe in their own words what they have done to control the NF. Strategy reports include the interpretation of experimenter’s instructions by participants and intentions to comply as well as the set of cognitive operations recognized by participants, which may manifest as trying to do nothing in particular, just letting things go.

We employed a classification key generated by Kober et al. (2013) to categorize mental strategies. A first aim of the present study is to investigate whether the training outcomes differ depending on the NF display. Another aim is to evaluate the objectivity of the classification key devised based on previous studies on mental strategies. A last aim of this study is to evaluate the frequency of occurrence and the effectiveness of mental strategies.

## Materials and Methods

### Sample

All individuals provided informed consent. The research was executed according to the guidelines of the Declaration of Helsinki. The study was approved by the Ethics committee of the University of Graz under GZ Nr. 13-2013/2014. Seventy-two individuals (38 women, 34 men, mean age = 22.75 years, SD age = 2.18 years) participated in the study. The participants were divided into two groups: One half (*n* = 36 people) completed a classic NF training using moving bars, while the other half (*n* = 36 people) tried the playful variant of a worm race. Individuals who have been diagnosed with psychiatric or neurological disorders during their lifetime were excluded from the study in advance. Similarly, head and neck surgery were exclusion criteria.

### Procedures

The measurements took place individually at the Institute of Psychology of the Karl-Franzens-University Graz. Participants were welcomed, introduced to the experimental setting and signed the informed consent. Participants filled out a demographic questionnaire and were then connected to the EEG system. Before the NF training, they completed two rest EEG measurements. These measurements lasted 2 min each and were performed once with eyes open and then with eyes closed. During the resting session with open eyes, participants were instructed to look at a fixation cross on a screen, relax and move as little as possible. After the rest measurements, participants were informed that they would now receive NF on their brain activity. The NF training was presented in two different ways: in the classic way using moving bars (see the section “Bar NF”) or as a serious game in form of a worm race (see the section “Worm Race”). The NF screens were presented in altering order, therefore the allocation of the participants to one of the two feedback screens can be considered as random. Detailed information about the NF training is given in section “SMR NF training.”

At the end, the participants wrote down which mental strategies they had used during NF training. The following instructions were presented to all participants: “*Please describe in a few words which strategies you have used during the neurofeedback training to control the bars.”* There were in total six different experimenters (5 women, 1 man), each one of them examined 12 persons (six using the bar NF and the other six using the worm race).

### SMR NF Training

The NF training was performed using the BioTrace + software (Mind Media B. V.) and consisted of a 3-min baseline measurement and the actual training (six feedback runs of 3 min each). The baseline measurement was used to determine the resting state of brain activity. Before this measurement, all participants were instructed to relax physically and do nothing while looking at the screen. Before the actual NF training began, all participants were instructed to focus on the task, stay physically relaxed, and not close their eyes. The specific instructions and procedures for both training screens are described below.

### Bar NF

**FIGURE 1:**
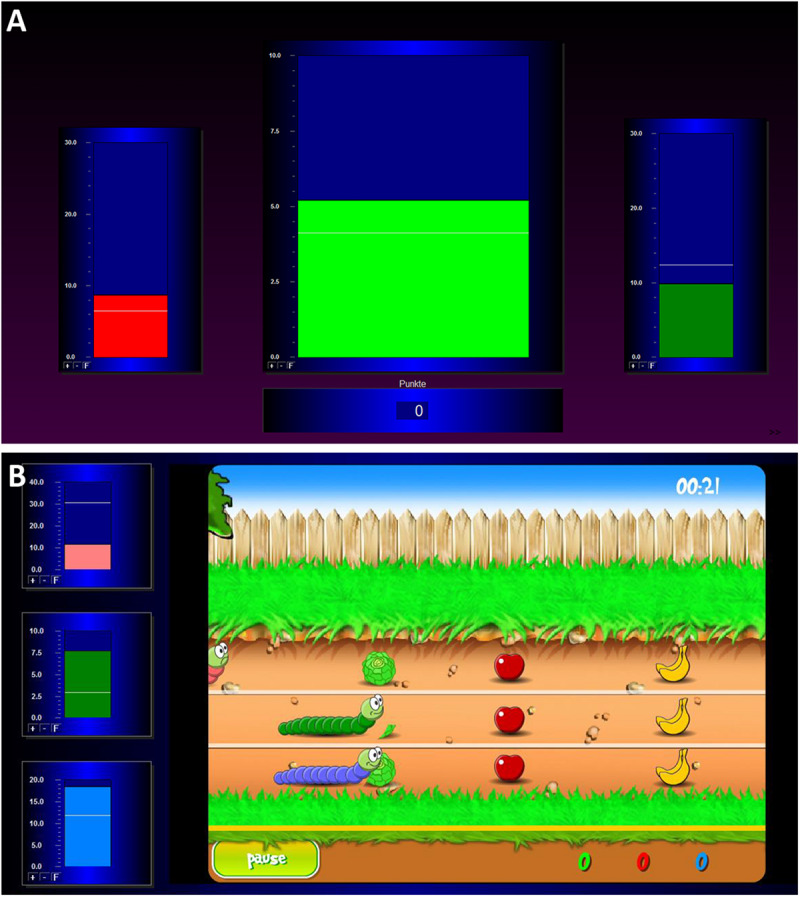
Illustration of the two feedback screens for the NF training. **(A)** The classic training, consisting of three bars which represent the EOG amplitude (left bar; 4–7 Hz), the SMR amplitude (middle bar; 12–15 Hz), and the muscle artifacts amplitude (right bar; 21–35 Hz). **(B)** The worm race, consisting of a racetrack and three worms reflecting the EOG, SMR, and muscle artifacts amplitudes on the left side of the screen.

During the baseline measurement, the participants saw three green bars moving up and down depicting the participants’ actual brain activity. After this measurement, the actual training began. During the individual feedback runs, three bars were visible on the screen again (see Figure 1A). The central bar of the feedback display showed the amplitude of the SMR activity. The right and the left bars reflected EEG activity in the theta and beta frequency ranges (EOG and muscle artifacts), respectively. Thresholds were automatically calculated for each participant and each run and were visualized as white horizontal lines. In the central bar, the threshold depicts the average power observed in the previous run. In the two lateral bars, the thresholds indicate the average power in these frequencies as observed during the baseline run +1 sd. Bar colors changed independently of each other depending on the power in the EEG frequency band it depicted. Participants were instructed to increase the size of the middle bar and to keep the size of the left and right bar constantly below their respective thresholds. Whenever the size of the SMR bar reached the predefined threshold, positive feedback was presented as a change of the bar color from red to green, in auditory format using a “Pling” sound, and as the number of points calculated based on the number of epochs fulfilling the criteria for positive feedback. There were no rewards, when the size of the EOG or muscle artifacts bar also reached the predefined thresholds. The goal was to score as many points as possible.

### Worm Race

During the baseline measurement, the participants saw the home screen of the game. The motionless picture showed the figures of the worm race. At the left side of the screen, three moving bars were shown, which moved up and down during the whole NF training depicting the participants’ actual brain activity. During training, the three worms moved from left to right (see Figure 1B) according to the brain activity of the participants. The movement of the green worm and the middle bar depicted the SMR amplitude. The movement of the pink worm and the upper bar showed the EOG artifacts (theta activity). The movement of the blue worm and the bottom bar reflected the muscle artifacts (beta activity). Thresholds were automatically calculated for each participant and each run in the same way as for the bar NF training. Now the instruction was to help the green worm to win the race. The worms moved forward as the associated bars crossed their respective white lines. Fruit and vegetables were also on the racetrack. When the worm ate the food, the participants were credited with points and eating noises. When the worm crossed the finish line, the participants were credited with additional points. There was a 10-s countdown before every race start. When the green worm reached 1000 points before the end of the 3 min, the worm turned into a butterfly and the race started again.

### Data Acquisition

The EEG was recorded using a NeXus-10 MKII (Mind Media B. V.) with a sampling rate of 256 Hz. The feedback electrode was placed over C3 (according to the 10-20 EEG placement system), the reference electrode behind the right mastoid, and one EOG channel placed over and under the left eye. The ground was located at the left mastoid. For the NF training, only the signal measured over C3 was used as feedback signal. The EEG signal was filtered in the SMR (12–15 Hz), theta (4–7 Hz), and beta (21–35 Hz) range using an IIR bandpass filter (Butterworth third order). Generally, eye movements lead to an increase in slower frequencies (e.g., theta, 4–7 Hz), where muscle activity leads to an increase in higher frequencies (e.g., beta, 21–35 Hz). The production of such artifacts also increases the amplitude of the EEG in SMR frequency range. To avoid participants to learn to manipulate the target feedback frequency by blinking their eyes or by contracting muscles voluntarily (Doppelmayr and Weber, 2011; Kober et al., 2013; Reichert et al., 2015), the frequency ranges of 21–35 Hz and 4–7 Hz also were considered when computing and presenting positive feedback. For feedback, the root-mean-square (RMS) was used to calculate the amplitude of the signal in the specific frequency ranges. The size of the epoch used to compute the RMS was 32 sampling points (125 ms). Positive feedback depended on the simultaneous fulfillment of three conditions: (1) power in beta frequency in the last epoch < mean beta power_baseline_ + 1sd beta power_baseline_, (2) power in theta frequency in the last epoch < mean theta power_baseline_ + 1sd theta power_baseline_, and (3) power in SMR frequency in the last epoch > mean SMR power_previous run_.

### EEG Data Analysis and Data Preparation

Following successful collection of the EEG data, EEG data preprocessing and analysis were performed with the Brain Vision Analyzer software (version 2.01, Brain Products GmbH, Munich, Germany). In this investigation, we only analyzed EEG data from the actual NF training, we didn’t analyze the resting state EEG.

Ocular artifacts such as eye blinks were manually rejected by visual inspection based on the information about EOG activity provided by the EOG channel. After ocular artifact correction, automated rejection of other EEG artifacts (e.g., muscles) was performed (criteria for rejection: >50.00 μV voltage step per sampling point, absolute voltage value > ±120.00 μV). All epochs with artifacts were excluded from the EEG analysis. For the EEG data analysis, absolute SMR (12–15 Hz) band power was extracted by means of complex demodulation (Brain Products GmbH, 2009). The power values were extracted and averaged over the whole artifact free training runs in one session. For statistical analyses and better comparability of the data, absolute SMR power values were z-transformed. With the standardized values, the slope of the regression line over all NF runs per participant was calculated. This value represents the average slope of SMR power across all passes, thus describing the average power or performance increase in low-frequency training.

### Categorization of Strategies

Individual strategies were assigned to one or more of nine different categories of responses defined according to Kober et al. (2013 and 2017): “Visual,” “Cheering,” “Breath,” “Auditory,” “Concentration,” “Body,” “Relax,” “Cognitive,” “No strategy,” “Other.” The category “Visual” describes all sorts of visual imagery, mental analogies to the bars or worms as well as descriptions of gaze direction and fixation at particular regions of the computer screen. “Cheering” describes a focus on any form of inner dialogue as well as voluntary efforts to motivate oneself, the bars or the worms to comply with the instructions. “Breath” describes the conscious breathing or the active control of breathing. “Auditory” strategies describe auditory imagery of pleasant music, tones or natural sounds. “Concentration” describes a deep and exclusive focus on the movement of the bars/worms as well as all degrees of perceived levels of concentration. “Body” describes the focus on the body activity or the activity of any of its parts: hands, legs, etc. It also subsumes descriptions of facial expressions, levels of tension in different body parts and a focus on the instructions not to move during training. “Relax” describes the feelings of turning off different body parts or the stream of thoughts, the search of a comfortable sitting position and relaxing of the face, neck and other body parts. “Cognition” subsumes the occurrence of thoughts, imagery, and memories not related to the task (e.g., the last date, the list of all US states, etc…). It also includes the explicit reference to positive and negative thoughts. “No Strategy” describes the explicit reference to not using any describable strategy to solve the task but rather just letting things flow at will. “Other” is the residual category to which any other non-classifiable strategy can be assigned. Depending on their complexity, individual strategies could be assigned to more than one category simultaneously.

In the present study, two raters were recruited. They were naïve regarding the research question as well as individual NF training outcomes, never faced participants undergoing NF training, and worked independently of each other. Raters were presented the strategy reports produced by each participant. Raters were then introduced to the classification key described above by MA and asked to rate, which categories occurred in the individual reports. Finally, they were instructed to employ as many categories as needed to describe the contents of individual strategy reports. Each rater generated a 62 × 9 matrix of binary values. The consistency of raters’ responses was established by means of the Kappa coefficient comparing the respective columns of the two matrices.

### Statistical Analysis

Estimating individual learning slopes and testing their consistency based on their variability penalizes for intraindividual inconsistencies and avoids the fallacy of significant average learning effects that do not apply to the majority of participants (Pinhas et al., 2012). For this reason, we obtained individual estimates of the learning slopes and tested them against interindividual slope variability. NF learning was defined as the linear slope of SMR power on training runs (baseline + 6 training runs). Participants were classified as responders or non-responders based on the sign of the regression slope. Participants with slopes ≤ 0 were classified as non-responders and participants with slopes > 0, as responders (i.e., the binary variable “SMR-responder”). The effects of NF screen, experimenter, as well as the interaction responder status vs. NF screen on learning outcomes were investigated in an ANCOVA model with age, sex, and experimenter identity as covariates.

We counted the frequency with which specific strategies were reported by individual participants (10 categories) as well as the frequencies of all possible pairings (9^∗^8/2 = 36 possible combinations, since the category “Other” was not detected by either of the raters). The frequency of pairs of strategies was also computed. It was defined as the percentage of the total number of strategy pairs given by each pair of strategies (i.e., the joint frequency of this strategy pair). Moreover, we analyzed the effectiveness of individual strategies as well as their pairings to upregulate the SMR rhythm. Effectiveness was defined as the average slope of those participants employing a specific strategy. Because most participants reported using more than one strategy, we also analyzed the effectiveness of pairs of strategies (i.e., joint effectiveness). Joint effectiveness was defined as the average slope observed among participants employing a given combination of strategies.

## Results

### NF Training Outcomes

**FIGURE 2:**
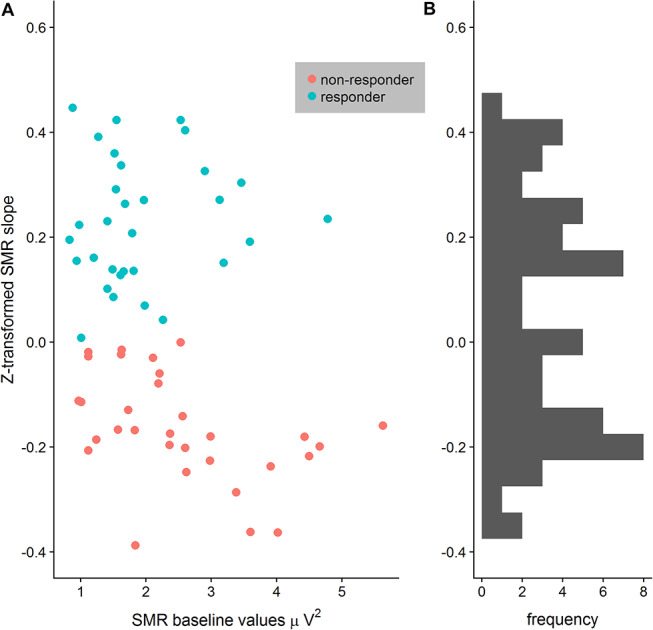
**(A)** Distribution of z-transformed SMR slopes depicted in function of SMR baseline values separately for responders and non-responders. The separation between responders and non-responders is particular clear when SMR baseline values increase. **(B)** Histogram of the z-transformed SMR slopes reveals no central tendency but rather a bimodal distribution.

**FIGURE 3:**
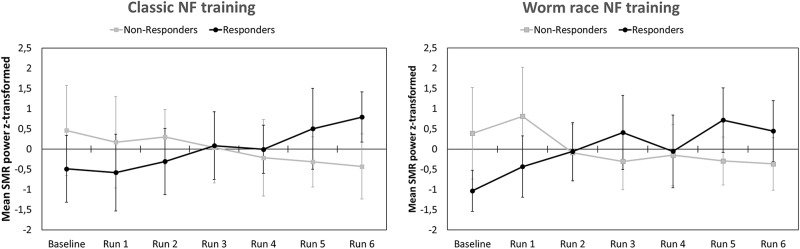
Means and standard deviations of z-transformed SMR (12–15 Hz) power (NF performance) for participants completing a classic NF training **(Left)** and participants completing a gamified NF training visualized by a worm race **(Right)**. NF performance is presented separately for responders and non-responders.

After EEG data analysis and data preparation, 13.9% of the participants were excluded from the sample: Two participants (2.8%) were excluded because of poor general EEG data quality, eight (11.1%) because of movement and muscle artifacts (more than 50% of EEG recording time had to be removed). The final sample consisted of 62 participants (29 women, 33 men, Mean age = 22.65 years, SD age = 2.19 years). Thirty-four persons completed the training in classic format and 28 persons as the worm race. Descriptive statistics are depicted separately for responders and non-responders trained either with the NF bars or the worm race scenario (Table 1). The proportion of non-responders did not differ between the classic format and the worm race (χ^2^ = 0.91, *df* = 1, *p* = 0.34). To investigate distribution of learning slopes of responders and non-responders, we depicted the individual SMR slopes in function of the SMR baseline values (Figure 2A) as well as a histogram (Figure 2B). The distribution of SMR slopes is not unimodal and shows a low density on 0, suggesting that the majority of participants tend to behave in a responder or in a non-responder mode. In an ANCOVA with feedback screen as a fixed-effect and sex, age, and experimenter as covariates, no main-effect of feedback screen was observed [*F*(1,58) < 1]. The effect of the covariate experimenter was close to significance (*p* = 0.07). In Figure 3, the NF performance is depicted for both types of feedback screens.

**TABLE 1:** Descriptive statistics for the z-transformed SMR slopes, sorted by responder group and type of feedback screen.

		Female/*N*	Age (years)	Mean	SD	Min	Max
*Responders (n* = *31)*	*Bar NF*	7/18	23	0.22	0.11	0.04	0.40
	*Worm race*	8/13	23	0.24	0.14	0.01	0.45
*Non-responders (n* = *31)*	*Bar NF*	5/16	23	−0.17	0.11	−0.39	0.00
	*Worm race*	8/15	22	−0.16	0.10	−0.36	−0.01

*Responders: Persons with an average slope > 0; Non-responders: Persons with an average slope ≥ 0.*

### Strategy Analysis

Frequency of occurrence of each strategy and the Kappa coefficient of concordance between raters are presented in Table 2. Concordance between raters was high or very high regarding the strategy categorization. The residual category “Other” was not employed by neither of the raters. In Table 2, frequency of occurrence is presented for the whole sample as well as separately for classic and worm race scenarios. Tests for the equality of proportions revealed no significant difference between both scenarios that were reproducible across raters in the ratings of single strategies (all *p*-values > 0.05).

**TABLE 2:** Frequency of occurrence of the different strategies.

	Strategy categories according to Kober et al. (2013)								No strategy	Other
	
	Visual	Cheering	Breath	Auditory	Concentration	Body	Relaxing	Cognition		
Rater 1	24/62	18/62	13/62	4/62	27/62	33/62	25/62	22/62	1/62	0/62
Bar NF	15/34	8/34	8/34	2/34	11/34	18/34	16/34	12/34	1/34	0/34
Worm race	9/28	10/28	5/28	2/28	16/28	15/28	9/28	10/28	0/28	0/28
Rater 2	25/62	18/62	13/62	5/62	29/62	32/62	26/62	22/62	1/62	0/62
Bar NF	16/34	8/34	8/34	2/34	12/34	18/34	16/34	12/34	1/34	0/34
Worm race	9/28	10/28	5/28	3/28	17/28	14/28	10/28	10/28	0/28	0/28
responders^§^	42%	50%	62%	50%	37%	45%	64%	64%	0%	–
Kappa	0.89	1.0	1.0	0.88	0.94	0.97	0.90	0.93	1.0	–

*^§^Probability of being a responder conditional on the employment of the respective strategy.*

**FIGURE 4:**
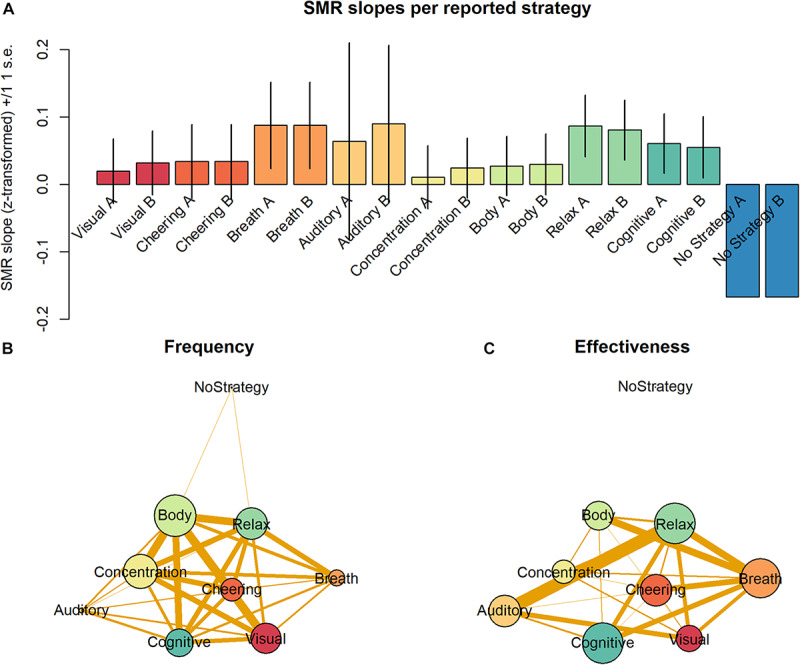
**(A)** Average SMR slopes are depicted for each one of the nine strategies as categorized by the two raters. **(B)** Frequency of occurrence of the nine different strategies is depicted by the vertex size. Edge thickness represents the probability of co-occurrence of the strategies. **(C)** Effectiveness of the nine different strategies as defined by the proportion of responders is depicted by the vertex size. Edge thickness represents the probability of co-occurrence of the strategies. Other parameters of the graphs **(B)** and **(C)** were arbitrarily defined for display purposes only.

Most strategies were used by more than 15% of all participants. Only 7% of all participants reported using one single strategy. Ordered by their frequency of occurrence, the strategies can be ranked as follows: “Body” > “Concentration” > “Relax” > “Visual” > “Cognition” > “Cheering” > “Breath” > “Auditory” > “No Strategy” (Table 2). When ordered by the proportion of responders using each strategy, the following ranking is observed: “Relax” = “Cognition” > “Breath” > “Auditory” = “Cheering” > “Body” > “Visual” > “Concentration” > “No Strategy” (Table 2). All strategies except for “No Strategy” were associated with a non-negative average slope (Figure 4A). The strategies “Breath,” “Cognitive,” and “Relax” were associated with SMR slopes larger than 0, what is indicative of efficient SMR power up-regulation. However, as indicated by the error bars, there is no evidence that some strategies are more efficient than other ones.

Importantly, participants reported on average the use of 2.7 strategies. This indicates that most participants employed between two to three strategies across the training session. To understand the effect of the combination of strategies, we analyzed the relative frequency with which pairs of strategies were combined in the individual reports. Figure 4B depicts the frequency of strategies as vertices and their joint frequency as edges. Vertex size represents the frequency of use of the individual strategies and the thickness of edges connecting the vertices depict the proportion of individual containing those two strategies. As “Body” was the most frequent strategy, its vertex is the largest. Interestingly, not only the edges connecting “Body” are thick and indicate frequent pairings but also many edges connecting the strategies “Concentration,” “Cheering,” “Visual,” and “Cognitive” have the same property.

Figure 4C depicts with vertex size the size of average slopes of individuals reporting those individual strategies. In contrast to Figure 4B, the vertex “Breath” is much larger in Figure 4C, for this strategy, although not very frequent, was particularly effective driving SMR up-regulation. The vertices representing “Relax” and “Cognitive” have a size comparable to that of “Breath,” for they equally belong to the most efficient strategies. In contrast, the vertices representing “Body” and “Concentration” are relatively small in Figure 4C, as they are not particularly efficient driving SMR up-regulation. Moreover, the edges connecting the vertices in Figure 4C depict the joint effectiveness of pairs of strategies. The most efficient pairs were “Relax” & “Auditory” and “Relax” & “Breath.” The strategy “Auditory” was rarely reported but very efficient. The strategy pair “Relax” & “Breath” also was particularly efficient. Interestingly, pairings with “Cognitive” seem to be slightly less efficient, although this strategy *per se* was very efficient. The majority of the pairings with “Cheering” and “Body” were quite frequent but showed very low effectiveness. Finally, “No Strategy” was a rare and very inefficient strategy to up-regulate the SMR rhythm, which was not paired with other strategies.

## Discussion

We investigated the association between mental strategies and SMR NF training outcomes. For this purpose, participants completed one session of NF training, where they were instructed to up-regulate the power in SMR frequency band (12–15 Hz). Two different feedback formats were employed, a classical and a gamified training. After concluding one single session of NF training, participants were asked to report their strategies to up-regulate SMR power, which were categorized by two independent raters. The concordance levels between the two raters was high for all strategy categories. The strategies most frequently used were “Body,” “Concentration,” and “Relax,” but the most efficient strategies were “Breath,” “Relax,” and “Cognitive.” Therefore, not necessarily the more frequent strategies are simultaneously the more efficient ones, what suggests that the degree of insight about the real effectiveness of mental strategies may be low when training SMR up-regulation for a single session. An effect of experimenter on training success was also observed. In the following sections, these results will be discussed in more detail.

### NF Training

The proportion of responders in the present study is largely comparable with other studies investigating the up-regulation of the SMR rhythm (Kober et al., 2013, 2017). After a single session of training, a large number of participants are non-responders since they still did not learn how to up-regulate the SMR rhythm. In the terminology of Gaume et al. (2016), limited training opportunity is probably insufficient to let participants develop a precise discrimination of internal states generating SMR upregulation. Due to the lack of discrimination, appropriate self-maintenance skills cannot develop either, and controlled SMR upregulation is not observed in non-responders. Therefore, one might argue that the categorization of participants as responders or non-responders after a limited amount of NF training is therefore artificial, particularly because a considerable number of participants may show a negative learning slope that is very close to 0. To investigate this possibility, we constructed a histogram of the learning slopes as well as a scatterplot of the learning slopes vs. the SMR power at the baseline (see Figure 3). The distribution of learning slopes was clearly not unimodal and showed a low density around the value 0. Hence, only a small number of participants showed small negative learning slopes. Moreover, the scatterplot suggests that the larger the baseline SMR power of a participant, the clearer is his/her status as a responder or non-responder. These results are in line with those by Reichert et al. (2015) regarding the discriminative value of baseline SMR power to separate responders from non-responders. One may speculate that low SMR power may require much higher discrimination skills (Gaume et al., 2016) than high power in this frequency band, so that participants with low SMR power have a disadvantage in comparison to individuals with higher baseline SMR power. Moreover, higher SMR power is not a sufficient condition for participants to upregulate the SMR rhythm, since there are non-responders with higher SMR baseline power as well. Future studies are necessary to disclose the role of frequency power in the individual ability to discriminate signals during NF training.

We replicated an effect of experimenter on the NF outcomes (Wood and Kober, 2018). The effect of experimenter was close to significance in the ANCOVA. This effect is not surprising, since in the last years, effects of experimenter on outcomes BCI and NF have been reported (Wood and Kober, 2018; Roc et al., 2019). For instance, Roc et al. (2019) observed an interaction between participants’ gender, experimenter gender, and progress over runs in a study with 59 participants. Moreover, in a study with 141 participants, Wood and Kober (2018) also found an effect of sex of experimenter and an interaction with the sex of participant. These results underscore the necessity to document and control for the effect of the interpersonal interaction on NF interventions (Chapman et al., 2018), since experimenter effects may be responsible for a substantial part of the confound effects generating the replicability crisis.

### NF Format and Gamification

The potential of game elements to improve NF has been investigated (Gilroy et al., 2013; Cavazza et al., 2014). The majority of studies on this topic reports proofs-of-principle, but a few studies have compared gamified and more traditional NF scenarios. For instance, Cohen et al. (2016) compared a simple uni-modal 2D interface with a more complex 3D scenario and found this to be more engaging and motivating than the 2D interface and to produce larger learning effects. Moreover, Gruzelier et al. (2010) argued that participants learned more efficiently to up-regulate the SMR when training in a 3D VR environment than with a traditional 2D computer screen, for a large between-groups difference emerged in the third session of training. Importantly, the advantage of the 3D VR environment observed in that study was compensated a few sessions later by the 2D NF. In the present study, feedback format had no effect upon the SMR slopes. Both the more traditional bars feedback as well as the worm-race were equally effective as a medium to provide NF to young adult and well-educated participants. Interestingly, participants receiving these two different feedback screens also reported largely the same mental strategies. One possible explanation for these results is that the graphical design of the worm-race does not suit well the game preferences of young and well-educated adults because the participant is forced to train using a worm-like avatar. Previous studies reveal that the avatar preferences observed among young adults are more diverse than in any other age group (Rice et al., 2013) and include non-human avatars. Therefore, no systematic negative effect of a worm avatar should be expected in our sample. Another possibility is that the fascination engendered by the connection of individual’s brain activity and visual feedback regardless of its more specific format is sufficient to produce high levels of engagement on NF training (Ali et al., 2014) and overshadow any positive or negative effects of gamification. Finally, it is possible that positive effects of gamification on motivation can be detected only after several sessions of training (Ninaus et al., 2015; Kober et al., 2016). When comparing two working memory training programs with and without game elements, Ninaus et al. (2015) observed the first positive effects of gamification on training engagement only after several sessions of training. The same phenomenon may occur during gamified versions of NF training.

### The Role of Independent Raters

At the initial stages of training, strategy reports may be considerably more complex than previous studies on SMR up-regulation may have suggested. As pointed out by Davelaar (2018), not all participants have the same subjective experience during NF training. Participants may experiment with a large diversity of strategies, which can be disentangled according to pre-specified classification systems. In the present study, the Kappa coefficients of inter-rater agreement show a high consistency among raters, who worked on the strategy reports independently from each other and were naïve to the experimental hypotheses tested in the present study. This can be considered evidence that the classification key is objective and largely independent from expectations and interpretation bias of experimenters (Shadish et al., 2002). The high concordance observed between independent raters suggests that the classification system generates reproducible results even when raters are blind to the identity of participants and the purposes of the experiment. Similar efforts may enrich the analysis of different types of NF intervention. In the case of SMR up-regulation training, the nine categories (Kober et al., 2013, 2017) seem to fulfill these criteria. The nine categories cover well the space of possibilities, so that in the present study the use of the residual category was not even necessary. Moreover, since the residual category “Other” was not employed a single time by the two raters, one may also conclude that the nine remaining categories of the classification key provide a largely exhaustive description of the mental strategies reported during SMR rhythm up-regulation.

### Frequency and Effectiveness of Individual Strategies

In the present study, the large majority of participants reported between two and three strategies (Figure 4B). These findings are compatible with the predictions of both models of NF learning (Gaume et al., 2016; Davelaar, 2018). As pointed out by Gaume et al. (2016), the development of the self-maintenance skills presupposes the participant to try several approaches to infer whether or not a strategy influences NF (Gaume et al., 2016). In a similar vein, the multistage theory of NF (Davelaar, 2018) expects participants to perform various mental acts and to evaluate their consequences on the feedback signal during the exploration stage of NF training.

We observed that many of the frequent strategies (“Body,” “Concentration,” and “Visual” with ranks 1, 2 and 4) occupy low ranks regarding their effectiveness (6, 8, and 7, respectively). In contrast, highly efficient strategies (“Relax, “Cognitive,” and “Breath,” with ranks 1, 2, and 3) occupy middle ranks regarding their frequency (3, 5, and 7). In previous studies, the authors have been inclined to interpret the frequent use of a strategy as evidence of its effectiveness (Nan et al., 2012). The present results indicate that the frequency of use of mental strategies is not necessarily an indicator of their effectiveness at least when training length is limited to one single session. In contrast, they reflect the fact that after a limited amount of training the approach of the large majority of participants is explorative. On the one hand, there is no reason to why participants should be able to start NF training with a good intuition about which strategy might be best to start training. Accordingly, among the more obvious strategies participants prefer at the start, some will reveal to be not particularly effective (Gaume et al., 2016). On the other hand, only exploration of one or more strategies can reveal more about their connection to the brain state participants desire to achieve to receive positive feedback (Gaume et al., 2016; Davelaar, 2018). Accordingly, only practice will reveal whether at a given timepoint the processes driving the modulation of desired brain states can be more easily driven by one instead of another strategy.

Except for the category “no strategy,” all strategies were relatively effective as participants used them to upregulate the SMR with some degree of success. Nevertheless, only the strategies “Breath,” “Relax” and “Cognitive” were associated consistently with slopes larger than 0 and therefore efficient for SMR power up-regulation. In our understanding, after a single session of SMR up-regulation training, it would be surprising, if specific strategies yielded results much superior to the average of all others, since at that point in time a precise interoceptive reference cannot be established.

In the present study, participants reporting the use of “No Strategy” showed poor performance. Only one single participant, who happened to be a non-responder, reported to have used “No Strategy” during training. Here, “No Strategy” may reflect lack of motivation, passivity, or even the belief that “doing nothing” is from the best strategy to learn to upregulate the SMR rhythm the beginning (which seems to be effective only after long practice). At the beginning of training participants cannot be enforced to a better performance by the use of strategies that typically will be efficient in later phases of training. When participants report “No strategy” after 10 sessions of training, they had practiced enough to develop a representation to aid self-regulation of the brain signals. In the first session of training, most participants do not have the time to develop a solid interoceptive representation. Moreover, they may have particular problems discriminating the internal states produced by SMR up-regulation, or are simply unable to effectively and volitionally self-regulate their internal states to upregulate the SMR rhythm reliably. For all these reasons, they benefit not from using “No strategy.”

### Joint Frequency and Effectiveness

As pointed out above, in the initial phases of SMR up-regulation participants tend to explore the use of more than one strategy and combined them. In the present study, the average participant reported two to three different strategy categories. To understand how the combination of strategies is related to the individual training outcomes, we investigated the joint frequency and joint effectiveness of all pairings of strategies reported by individual participants. The joint frequency of most strategies showed similar properties. The vertices connecting the strategies “Body,” “Concentration,” “Cheering,” “Visual,” and “Cognitive” revealed frequent pairings between all these strategies and are suggestive of the exploratory character of participants attitude during training. Considering that participants reported on average 2.7 strategies and only 7% of all participants reported using only one strategy, thoroughly exploring the space of possibilities is in line with the multistage theory (Davelaar, 2018) as well as with the psychoengineering paradigm (Gaume et al., 2016).

Analysis of the joint effectiveness of strategies also revealed that some strategies such as “Breath” and “Relax” present mainly highly efficient pairings (thick vertices), while “Cognitive” and “Cheering” present mainly inefficient pairings. The vertices representing “Relax” and “Cognitive” have a size comparable to that of “Breath,” for they equally belong to the most efficient strategies. These results can be interpreted as evidence that strategies such as “Breath” and “Relax” support the use of other strategies, and others such as “Cognitive” do not work efficiently in combination with others except “Breath” and “Relax” (Figure 4C). Together, these explorative results are suggestive about similarities in the internal representations underlying some but not all efficient strategies and the self-maintenance required by the different strategies (Gaume et al., 2016). Moreover, they underscore the need to perform a detailed analysis of the subjective experience of participants (Davelaar et al., 2018) with the aim of optimizing NF training.

Previous studies indicate that giving detailed and specific strategies to participants at the beginning of training may be counterproductive (Hardman et al., 1997) and that it is preferable to keep instructions simple enough not to bias the learning process in a negative way. However, with a better understanding of the space of possibilities contained in strategy reports, it may be possible to keep initial instructions simple while integrating complementary feedback on the choice of strategies in the NF protocol as participants report them during or at the end of training sessions. One practical advice one can take from the present findings is to avoid mentioning the concept of “concentration” in the instructions as well as the occurrence of “No Strategy” in later stages of training, because they may hamper learning in the early phases of a more sessions training program.

Categorization by raters seems to be useful to describe the kind of mental image utilized by each participant and the intermingling of different types of mental images. Therefore, outcomes of categorization by raters can be useful to guide participants across training sessions into approaching or avoiding certain types of mental imagery depending on their outcomes. For instance, participants reporting frequent but inefficient strategies may be encouraged by the therapist to try a more effective one. The detailed analysis of strategy reports in light of pre-specified classification procedures which have been validated by evidence may help to optimize training protocols and improve NF outcomes. To fully exploit and use the information stored in human language, one should consider the use of more innovative methods to evaluate mental strategies. This could be done by using a phenomenological approach (see Davelaar et al., 2018) or a more economical way, for example automatic vocabulary classification.

## Data Availability Statement

The datasets generated for this study are available on request to the corresponding author.

## Ethics Statement

The studies involving human participants were reviewed and approved by the Ethics Committee of the University of Graz under GZ Nr. 13-2013/2014. The patients/participants provided their written informed consent to participate in this study.

## Author Contributions

MA collected and analyzed text data and wrote the manuscript. SK analyzed EEG data. CN read and commented the study. GW conceived the study, analyzed data, and wrote the manuscript.

## Conflict of Interest

The authors declare that the research was conducted in the absence of any commercial or financial relationships that could be construed as a potential conflict of interest.
